# From conceptual emergence to multidisciplinary expansion: a bibliometric and knowledge mapping analysis of cardiovascular–kidney–metabolic syndrome research

**DOI:** 10.3389/fendo.2026.1895884

**Published:** 2026-07-28

**Authors:** Tian Lv, Yu-Jun Xiong, Qian-Yuan Zhu

**Affiliations:** 1 Department of Neurology, Zhuji Affiliated Hospital of Wenzhou Medical University, Zhuji, China; 2 Department of Gastroenterology, Beijing Hospital, National Center for Gerontology, Beijing, China; 3 National Clinical Research Center for Gerontology, Beijing, China; 4 The Key Laboratory of Geriatrics of NHC, Beijing, China; 5 Institute of Geriatric Medicine, Chinese Academy of Medical Sciences, Beijing, China; 6 Department of Neurology, Fenghua Hospital of Traditional Chinese Medicine, Ningbo, China

**Keywords:** bibliometric analysis, biblioshiny, CiteSpace, CKM, VOSviewer

## Abstract

**Background:**

The concept of cardiovascular-kidney-metabolic (CKM) syndrome has recently been introduced as an integrated framework linking heart, kidney, and metabolic disorders. However, a global overview of the research landscape in this emerging field is still lacking.

**Methods:**

Publications related to CKM published between 2023 and 2025 were retrieved from the Web of Science Core Collection and Scopus. The search strategy incorporated the following terms: TS = (“cardiovascular-kidney-metabolic” OR “cardiovascular kidney metabolic” OR “cardiovascular-kidney-metabolic syndrome” OR “cardiovascular kidney metabolic syndrome” OR “CKM syndrome” OR “CKM health” OR “CKM framework” OR “cardiovascular-kidney-metabolic health”). Bibliometric indicators, including publication volume, authorship patterns, institutional and country contributions, journal distribution, co-citation networks, and keyword burst analysis, were systematically assessed.

**Results:**

A total of 1, 854 articles from 665 journals were identified. Annual output surged from 8 in 2023 to 1, 544 in 2025. A total of 8, 359 authors contributed, with an average of 7.95 co-authors per paper, reflecting strong collaborative activity. China and the United States were the most productive countries, with the latter acting as a central node in international collaboration networks. Keyword and citation analyses showed a clear shift from individual risk factors toward integrated cardiometabolic-renal interactions, clinical outcomes, and mortality. Highly cited works mainly addressed the definition, epidemiology, and clinical relevance of CKM.

**Conclusion:**

Since its introduction in 2023, CKM research has grown rapidly into a collaborative and multidisciplinary field. Future efforts should focus on strengthening international partnerships, advancing mechanistic research, and improving the clinical translation of the CKM framework for risk stratification and prevention.

## 1 Introduction

Cardiovascular-kidney-metabolic (CKM) syndrome has recently emerged as an important multidimensional concept that integrates the complex interactions among cardiovascular disease, chronic kidney disease, metabolic disorders, and obesity-related conditions (1). In 2023, the American Heart Association formally defined CKM syndrome to address the shared pathophysiological mechanisms—including insulin resistance, chronic inflammation, oxidative stress, endothelial dysfunction, and neurohormonal activation—that link these conditions (2).

Unlike traditional models that assess cardiovascular, renal, and metabolic diseases separately, the CKM framework acknowledges their frequent coexistence and bidirectional interactions. This shift is clinically relevant given the rising global prevalence of obesity, type 2 diabetes, hypertension, and chronic kidney disease. Individuals with overlapping abnormalities face substantially higher risks of myocardial infarction, stroke, heart failure, end-stage renal disease, and premature death than those with a single condition (3). The dramatic increase in obesity and diabetes worldwide has further accelerated CKM-related complications (4). Importantly, metabolic dysfunction and kidney impairment often precede overt cardiovascular disease, underscoring the need for early, integrated risk stratification (5). Compared with earlier concepts such as metabolic syndrome or cardiorenal syndrome, CKM adopts a broader life-course perspective emphasizing cumulative, multidirectional organ interactions.

Since 2023, research on CKM syndrome has grown rapidly, covering epidemiology, biomarkers, prevention, and treatments—particularly sodium-glucose cotransporter-2 inhibitors, glucagon-like peptide-1 receptor agonists, obesity management, and precision medicine (6). Social determinants of health and health inequities have also gained attention as key modifiers of CKM outcomes (7). However, no comprehensive bibliometric analysis has mapped the overall research landscape, collaboration networks, influential publications, or emerging hotspots in this field. Accordingly, this study aimed to systematically analyze global research trends in CKM syndrome, identify major contributors and collaborative networks, and explore evolving frontiers since the concept’s introduction.

## 2 Materials and methods

### 2.1 Data sources

**Figure 1:**
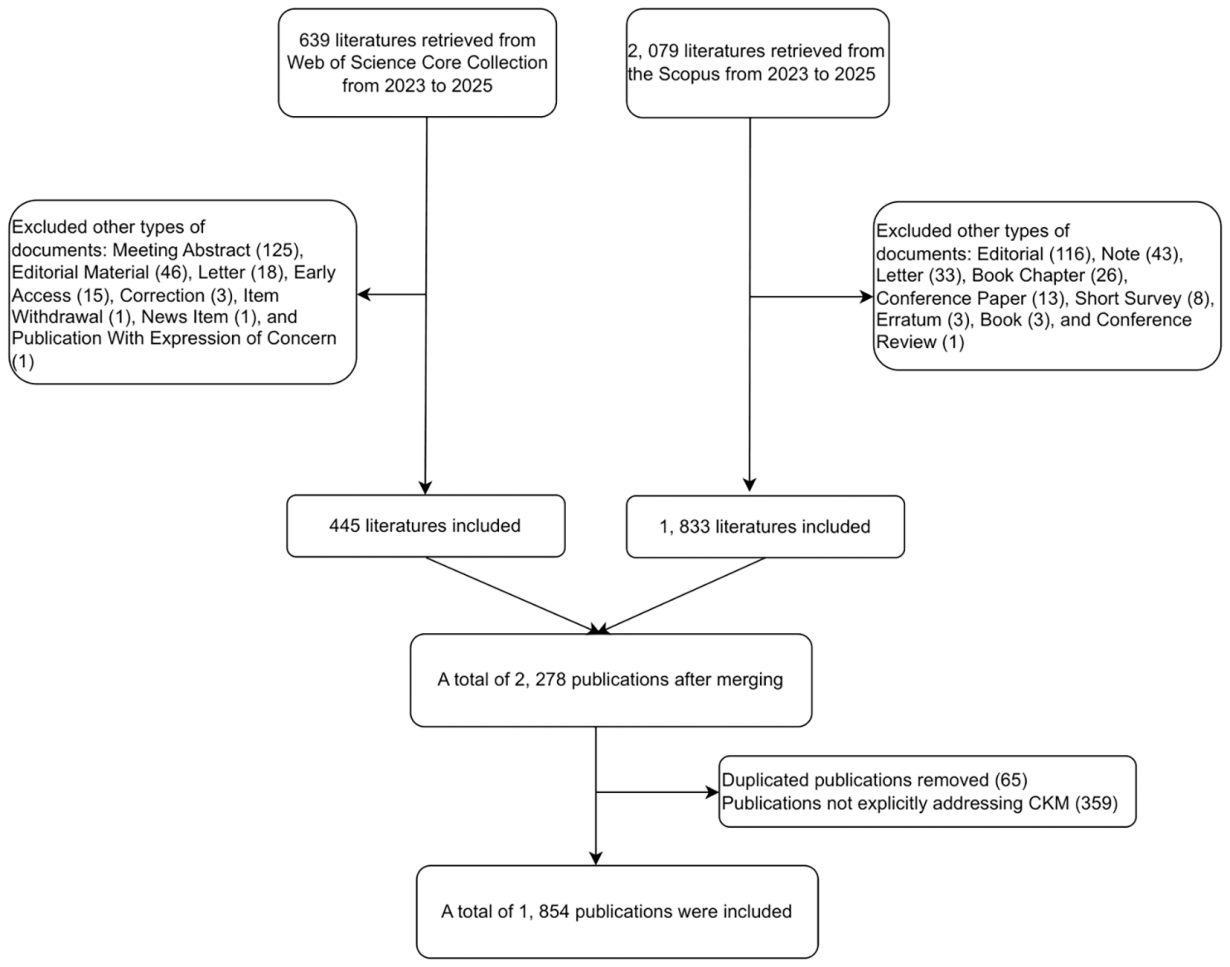
Flow chart of literature filtering.

To capture relevant studies published between January 2023 and December 2025, we conducted a comprehensive literature search using the Science Citation Index Expanded (SCI-Expanded) of the Web of Science Core Collection and the Scopus database. The search was performed on May 20, 2026, to minimize potential bias from subsequent database updates. The following search string was applied in the Web of Science Core Collection database: TS = (“cardiovascular-kidney-metabolic” OR “cardiovascular kidney metabolic” OR “cardiovascular-kidney-metabolic syndrome” OR “cardiovascular kidney metabolic syndrome” OR “CKM syndrome” OR “CKM health” OR “CKM framework” OR “cardiovascular-kidney-metabolic health”). In Scopus, the equivalent search was performed using the TITLE-ABS-KEY field with the same search terms. Eligible records were restricted to original research articles and review articles published in English. Studies investigating cardiovascular disease, chronic kidney disease, diabetes, obesity, metabolic syndrome, or related conditions without explicitly addressing CKM were excluded. A total of 445 records were retrieved from the Web of Science Core Collection and 1, 833 records from Scopus. Non-research outputs, including conference proceedings, editorials, meeting abstracts, and other document types, were excluded. After document type and language filtering, the records from both databases were merged using the Biblioshiny interface of the Bibliometrix R package. Duplicate records were identified and removed based on DOIs, article titles, and author names, resulting in a final dataset of 1, 854 publications for bibliometric analysis. The complete study selection process is illustrated in **Figure 1**.

### 2.2 Data collection

Initial records were retrieved from multiple literature databases, including Scopus and the SCI-Expanded of the Web of Science Core Collection. Records exported from both databases were imported into the Biblioshiny interface of the Bibliometrix R package, where they were merged into a single bibliographic dataset. Duplicate records were identified and removed based on DOIs, article titles, and author names before screening. The deduplicated merged dataset was subsequently used for all bibliometric analyses performed in Bibliometrix/Biblioshiny, VOSviewer, and CiteSpace, ensuring consistency across all analyses. Two reviewers (TL and YJX) independently assessed all identified publications to determine their relevance to the research theme based on titles and abstracts. Full-text review was performed when necessary to determine whether the CKM concept represented the primary focus of the publication. The screening process was carried out primarily by evaluating titles and abstracts, with full-text reviews conducted when needed. Disagreements between the two reviewers were resolved through discussion until a consensus was reached. Following the screening, bibliographic data—such as publication year, authors, countries, institutions, journals, keywords, and cited references—were extracted for subsequent analysis.

### 2.3 Statistical analysis

To analyze publication characteristics such as annual output, citation performance, country and institutional contributions, journal distribution, and impact factors, we used Microsoft Excel 2019 along with the Bibliometrix package in R. Keyword clustering, temporal evolution, and burst detection were carried out using CiteSpace. The parameter settings covered the period from 2023 to 2025, with one-year time slices, a g-index selection criterion (k = 25), and Pathfinder network pruning. For visualizing collaboration networks among countries, institutions, and authors, as well as keyword co-occurrence, we used VOSviewer (version 1.6.18). Specifically, VOSviewer analyses were performed using the full counting method with a minimum document threshold of 25 and a minimum citation threshold of 0. CiteSpace (version 7.0.1) was used for keyword burst detection and reference citation burst analysis. The parameter settings included a time span of 2023–2025, one-year time slices, the g-index selection criterion (k = 25), and Pathfinder pruning to simplify the generated networks. This step helped uncover research links and cooperation patterns across different groups. Statistical work and figure generation were done using R software (version 4.3.2). Bibliometrix was used to show publication trends over time, while Biblioshiny (version 4.3.2) supported keyword clustering and thematic mapping. Combining these tools gave us a broad view of the research landscape, emerging topics, and the overall knowledge structure in this field.

## 3 Results

### 3.1 An overview of publications

Between 2023 and 2025, a total of 1, 854 publications related to CKM were identified from the selected literature sources. These studies were distributed across 665 journals and related sources. Publication output increased markedly from 2023 to 2025, reflecting growing research interest in this emerging field. Given the very small number of publications in 2023, the calculated annual growth rate should be interpreted cautiously. The average citation count per document was 11.22, supported by a total of 18, 921 cited references, indicating a growing but still developing body of evidence. A total of 8, 359 authors contributed to the publications, highlighting the broad academic engagement in CKM research. Regarding authorship characteristics, 72 publications were completed by a single author, whereas the average number of co-authors per article was 7.95, demonstrating a strong tendency toward collaborative research. In addition, international collaborations accounted for 4.75% of all publications, suggesting that cross-national academic cooperation has gradually emerged in this field.

### 3.2 The annual publications of CKM

**Figure 2:**
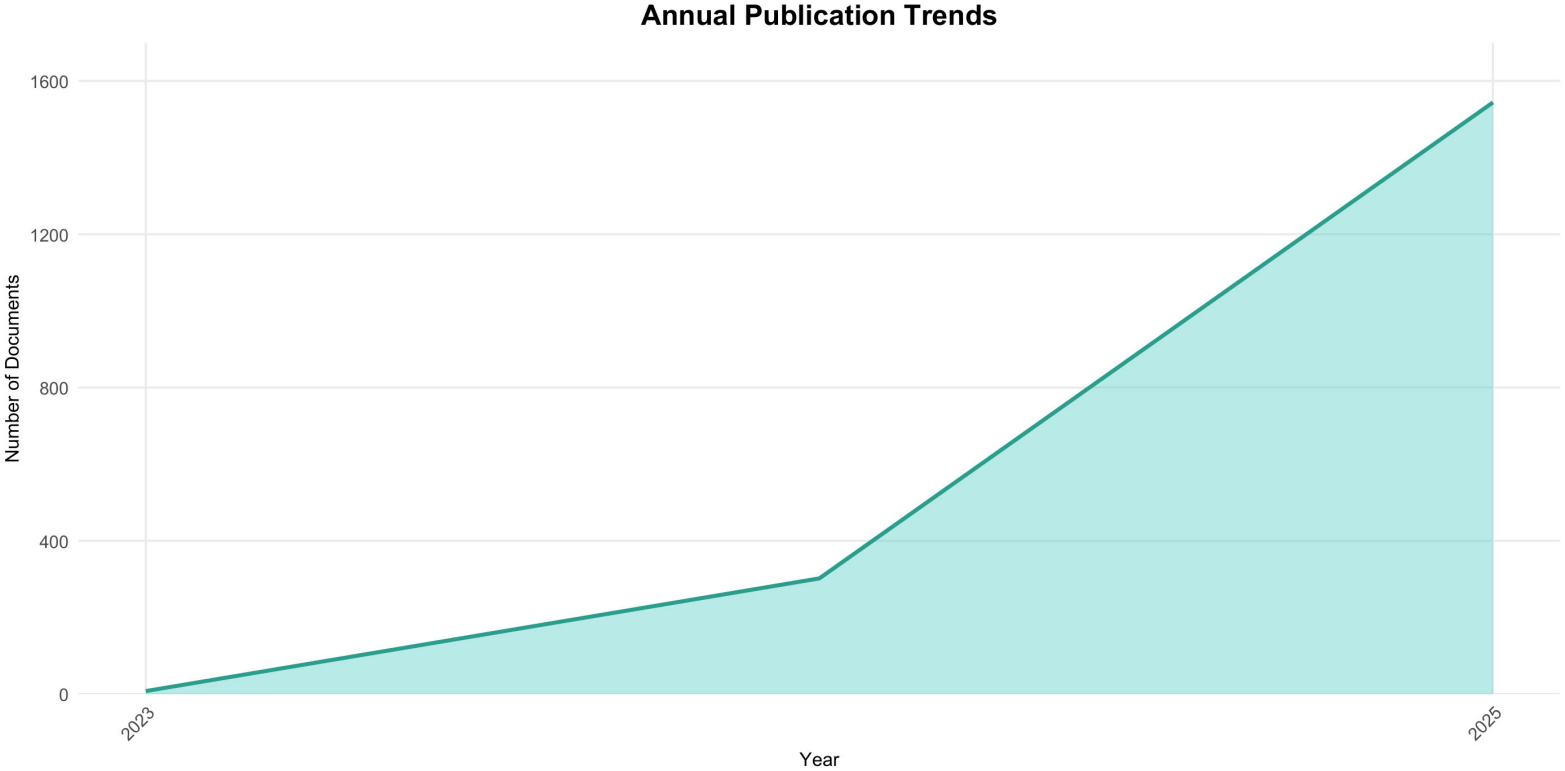
The number of annual publications.

As illustrated in **Figure 2**, the annual number of publications on CKM demonstrated a dramatic upward trend between 2023 and 2025. The number of publications increased sharply from 8 in 2023 to 302 in 2024, and further surged to 1, 544 in 2025. This rapid expansion in research output highlights the growing scientific interest in CKM and reflects increasing attention to its epidemiological burden, pathophysiological mechanisms, risk assessment, and clinical management.

### 3.3 The contributions and collaborations of countries and institutions

**Figure 3:**
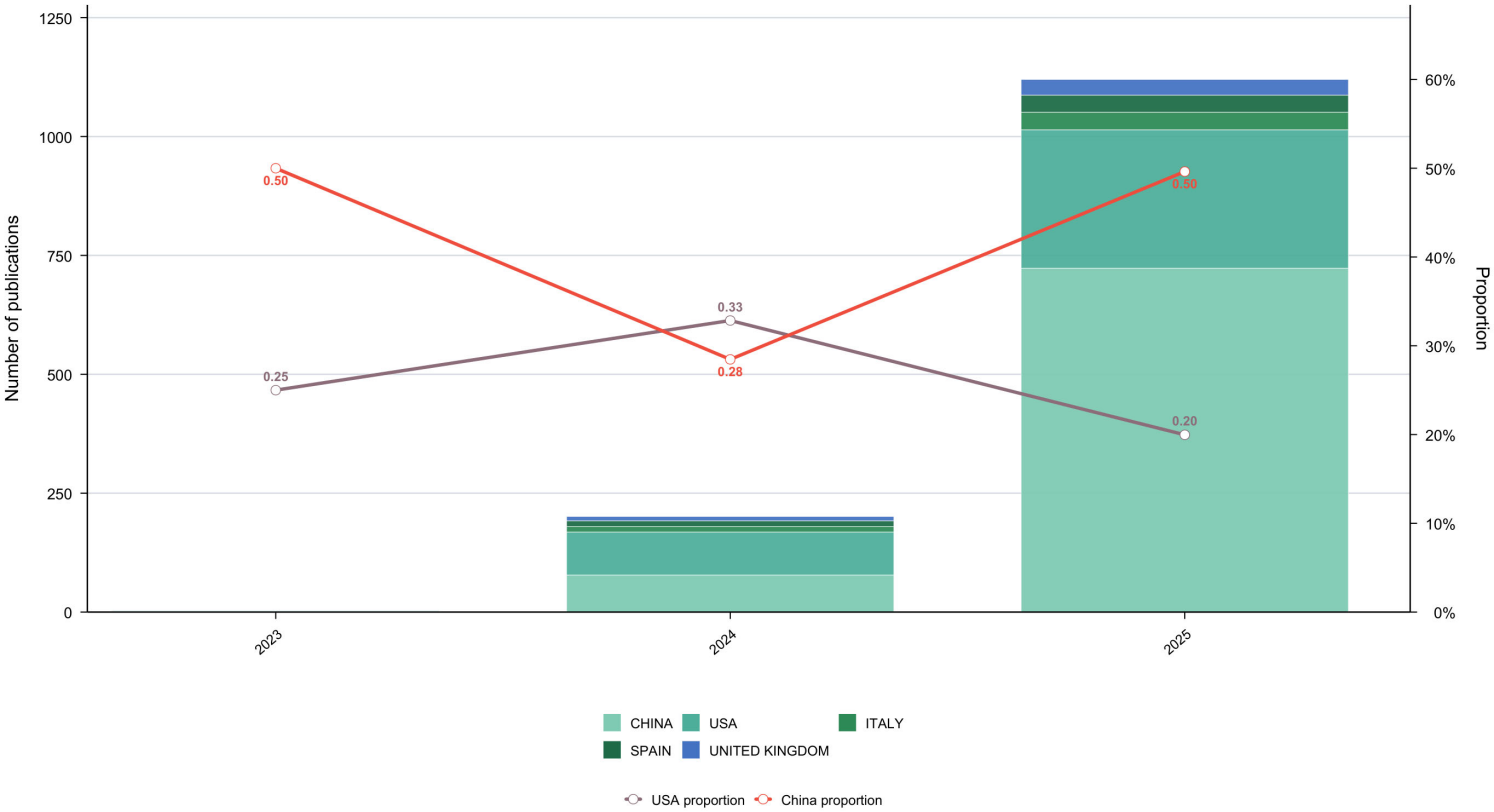
Growth trends of the top 5 countries.

Over the past three years, China has consistently been a major contributor to CKM research, followed by the United States, Italy, Spain, and the United Kingdom (**Figure 3**). Although China led in both 2023 and 2025, the United States ranked first in the number of publications in 2024. Despite these yearly shifts, China remained the dominant force in the field over the entire study period.

**Figure 4:**
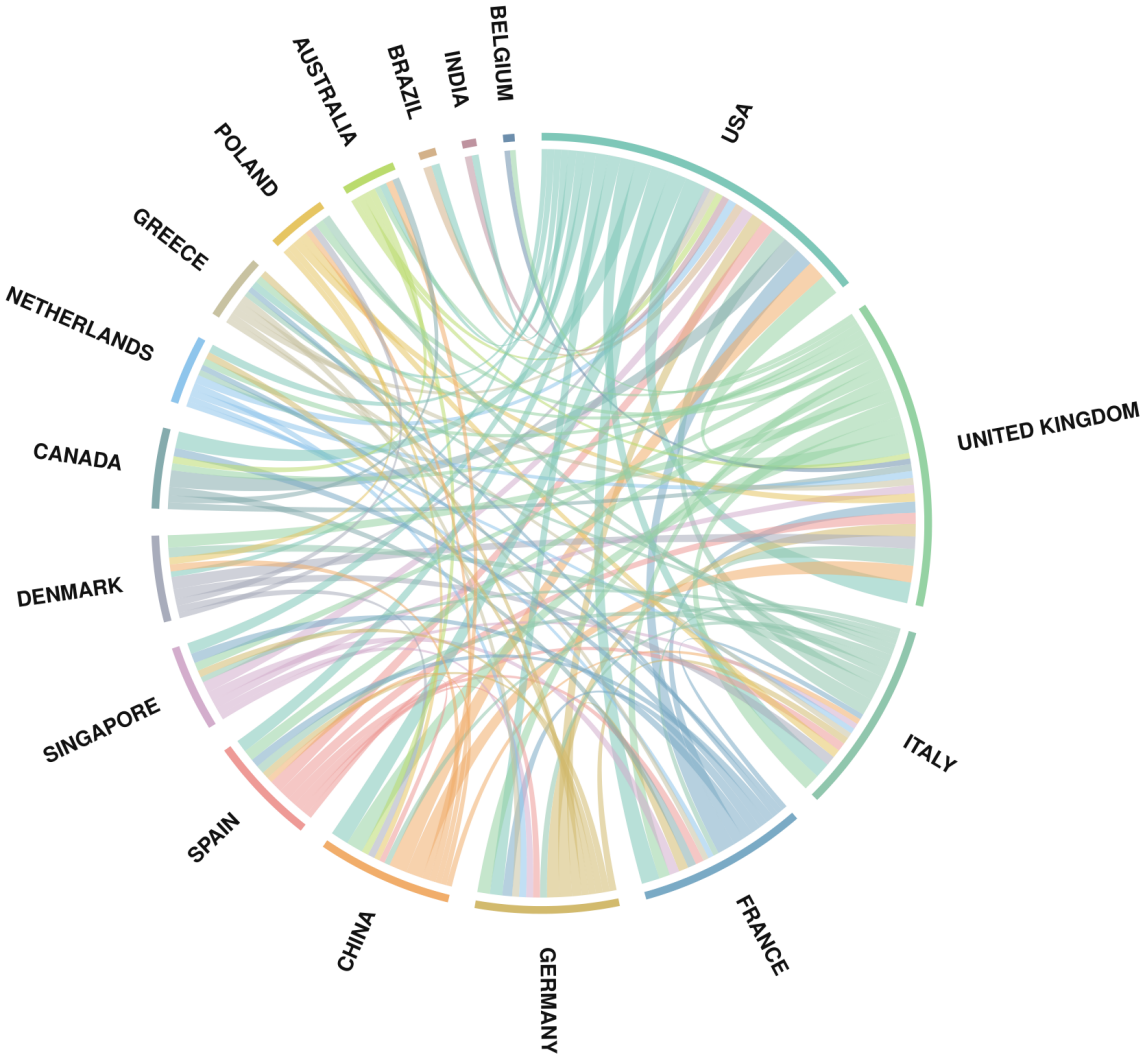
The distribution of countries/regions. Cooperative relations among countries/regions (The area represents the number of articles, and the connection represents the cooperative relationship. Conducted by online analysis platform of Bibliometrics).

**Figure 5:**
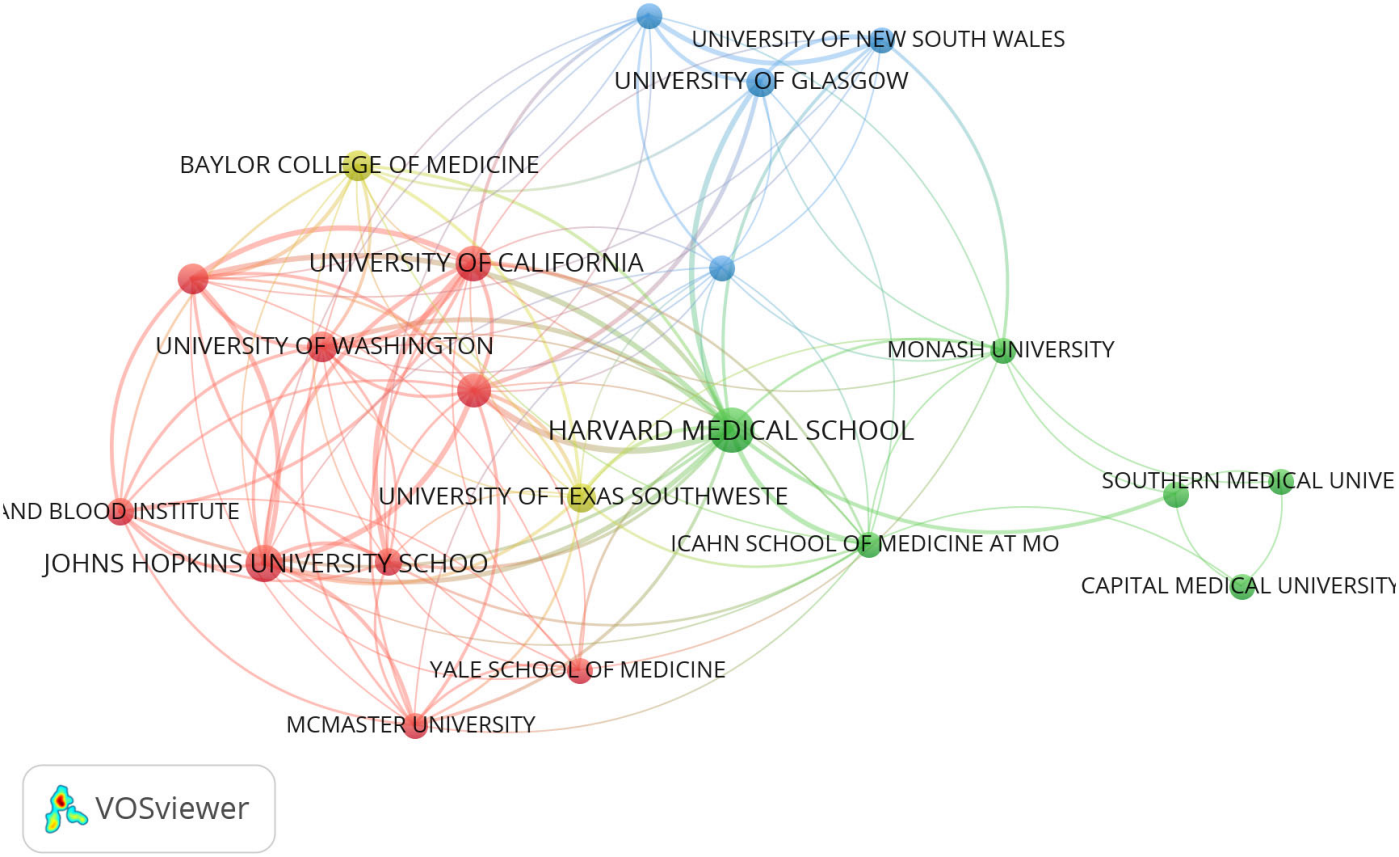
The distribution of institutions. Cooperative relations among institutions (The area represents the number of articles, and the connection represents the cooperative relationship. Conducted by VOSviewer).

An analysis of international collaboration revealed that the United States served as the central hub of global cooperation, with the strongest ties to the United Kingdom, Italy, France, Germany, and China. European countries such as Germany, Italy, France, and Spain also formed dense collaborative networks. In Asia, China showed growing international involvement, particularly with the United States, the United Kingdom, and Canada (**Figure 4**). As shown in **Table 1**, China produced the most publications in CKM-related research, with 803 articles (54.96%), followed by the United States (382, 26.15%), Italy (49, 3.35%), Spain (48, 3.29%), and the United Kingdom (43, 2.94%). Australia, South Korea, Canada, Greece, and Japan also made significant contributions. On the institutional level, Harvard Medical School was the most productive, with 64 papers, followed by Capital Medical University (59), the University of California (58), Southern Medical University (53), and the Chinese Academy of Medical Sciences & Peking Union Medical College (49). Other key contributors included Capital Medical University, Kaohsiung Chang Gung Memorial Hospital, Johns Hopkins University School of Medicine, Northwestern University Feinberg School of Medicine, and Nanjing Medical University. As seen in **Figure 5**, strong collaborative links existed among the leading research institutions.

**Table 1:** The top 10 countries/regions and institutions contributing to publications in cardiovascular-kidney-metabolic syndrome related research (sorted by count).

Rank	Country	Article counts	Percentage	Institutions	Article counts	Percentage
1	CHINA	803	54.96%	HARVARD MEDICAL SCHOOL	64	13.47%
2	USA	382	26.15%	CAPITAL MED UNIV	59	12.42%
3	ITALY	49	3.35%	UNIVERSITY OF CALIFORNIA	58	12.21%
4	SPAIN	48	3.29%	SOUTHERN MED UNIV	53	11.16%
5	UNITED KINGDOM	43	2.94%	CHINESE ACAD MED SCI AND PEKING UNION MED COLL	49	10.32%
6	AUSTRALIA	38	2.60%	CAPITAL MEDICAL UNIVERSITY	43	9.05%
7	KOREA	31	2.12%	KAOHSIUNG CHANG GUNG MEM HOSP	40	8.42%
8	CANADA	27	1.85%	JOHNS HOPKINS UNIVERSITY SCHOOL OF MEDICINE	37	7.79%
9	GREECE	20	1.37%	NORTHWESTERN UNIVERSITY FEINBERG SCHOOL OF MEDICINE	37	7.79%
10	JAPAN	20	1.37%	NANJING MED UNIV	35	7.37%

### 3.4 Research productivity by journals and authors

Between 2023 and 2025, a total of 665 journals published CKM-related studies. Of the 1, 854 articles included, the top ten journals contributed 365 papers, accounting for 19.69% of all retrieved publications (**Table 2**). Cardiovascular Diabetology published the most articles on CKM, followed by Frontiers in Endocrinology and Frontiers in Nutrition. According to the 2023 Journal Citation Reports, most of these journals were in the Q1 category, with impact factors ranging from 2.6 to 10.1.

**Table 2:** The top 10 most active journals published articles in cardiovascular-kidney-metabolic syndrome related research (sorted by count).

Rank	Journal title	Article counts	Percentage	IF	JCR
1	CARDIOVASCULAR DIABETOLOGY	67	18.36%	10.6	Q1
2	FRONTIERS IN ENDOCRINOLOGY	52	14.25%	4.6	Q1
3	FRONTIERS IN NUTRITION	52	14.25%	5.1	Q1
4	SCIENTIFIC REPORTS	36	9.86%	3.9	Q1
5	INTERNATIONAL JOURNAL OF MOLECULAR SCIENCES	32	8.77%	4.9	Q1
6	JOURNAL OF CLINICAL MEDICINE	32	8.77%	2.9	Q1
7	BMC PUBLIC HEALTH	24	6.58%	3.6	Q1
8	JACC: ADVANCES	24	6.58%	0	/
9	AMERICAN JOURNAL OF PREVENTIVE CARDIOLOGY	23	6.30%	5.9	Q1
10	PLOS ONE	16	8%	2.6	Q2

**Figure 6:**
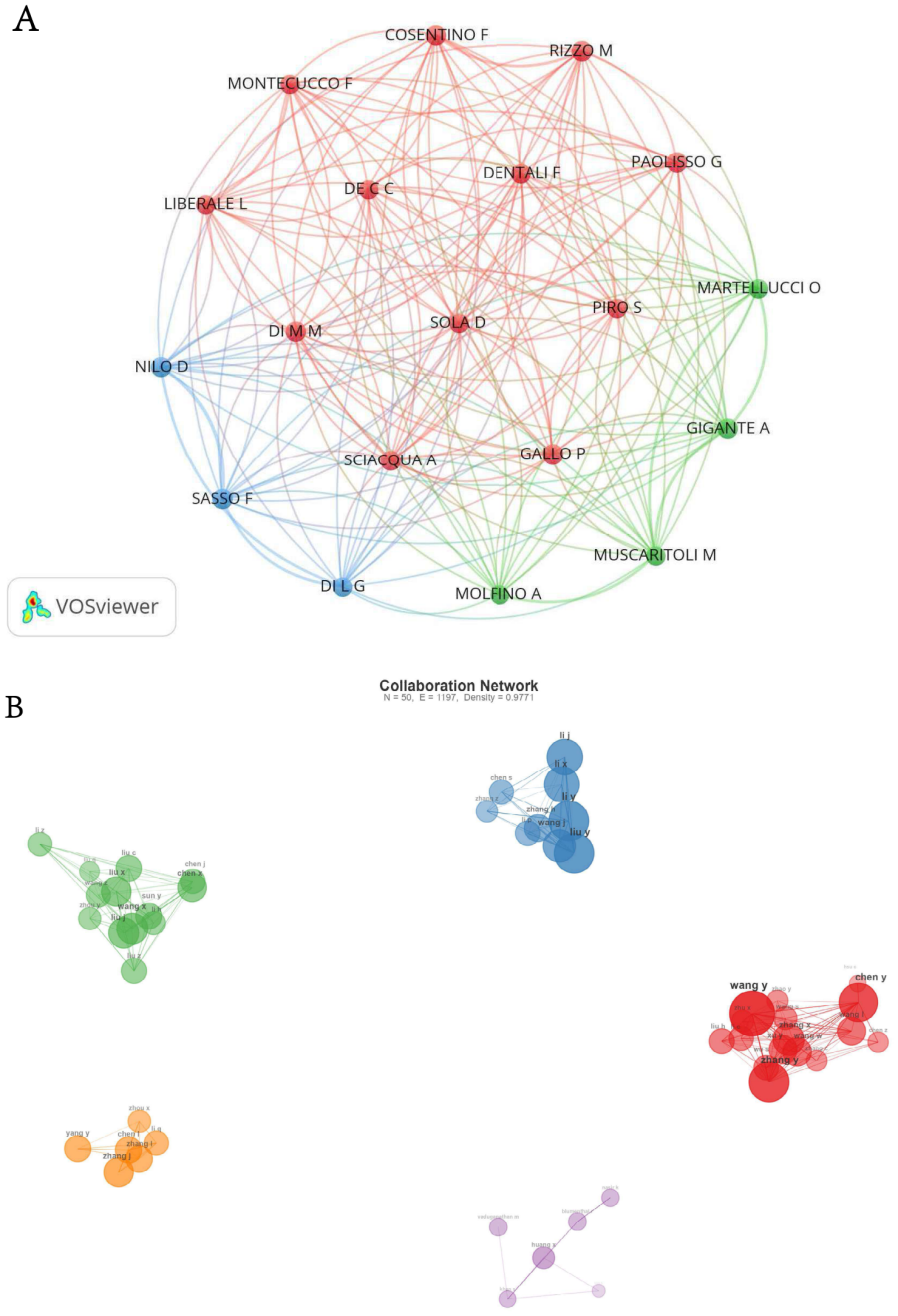
Cooperation map of authors in the studies of CKM. **(A)** Conducted by VOSviewer; **(B)** Conducted by Biblioshiny R package.

**Figure 7:**
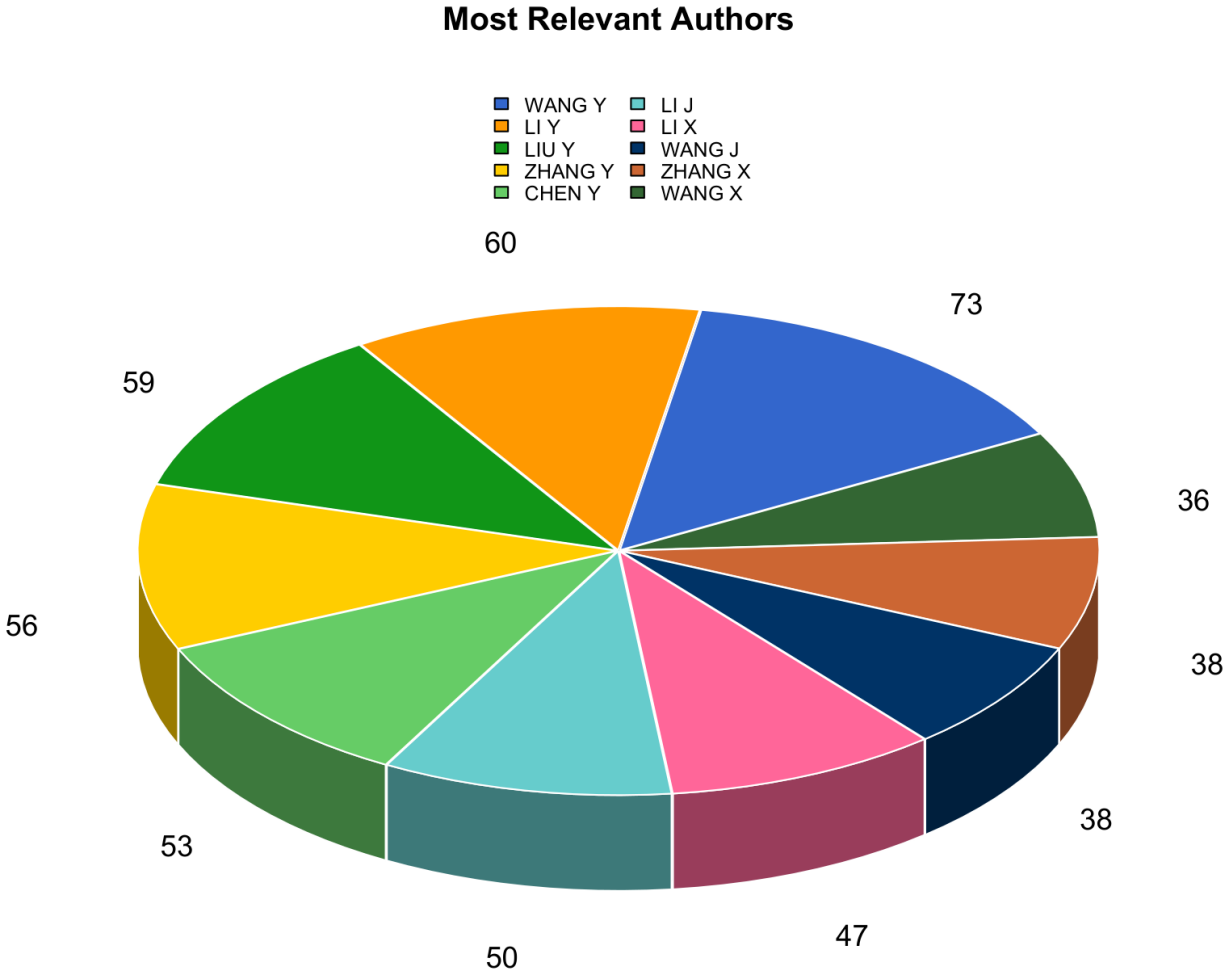
Most relevant authors in the field of CKM.

**Figures 6A, B** illustrate the author collaboration network in CKM research, revealing several distinct clusters of closely linked researchers. For instance, SOLA D worked closely with DENTALI F and GALLO P, while WANG Y frequently collaborated with ZHANG Y, indicating stable research partnerships in this field. **Figure 7** highlights the most productive authors in CKM research over the past three years. WANG Y ranked first with 73 publications, followed by LI Y (60), LIU Y (59), and ZHANG Y (56). Other notable contributors included CHEN Y and LI J. Collectively, these authors have played key roles in advancing CKM research.

Lotka’s law analysis demonstrated that author productivity in CKM research was highly skewed (**Table 3**). Among all authors, 6, 459 (77.3%) published only one article, exceeding the theoretical proportion predicted by Lotka’s law (61.8%). The proportion of authors declined rapidly as publication output increased, with only 10.8% publishing two articles and 4.1% publishing three articles. These findings indicate that the CKM field is currently dominated by occasional contributors, whereas a relatively small number of authors have established sustained research productivity.

**Table 3:** Distribution of author productivity according to Lotka’s law.

Documents written	Number of authors	Proportion of authors	Theoretical
1	6459	0.773	0.618
2	899	0.108	0.154
3	339	0.041	0.069
4	202	0.024	0.039
5	118	0.014	0.025
6	81	0.01	0.017
7	51	0.006	0.013
8	39	0.005	0.01
9	26	0.003	0.008
10	22	0.003	0.006

### 3.5 Co-cited documents and journals

We summarized the top 10 most cited CKM articles published between 2023 and 2025 in **Table 4**, including details such as first author, journal, DOI, and citation counts for assessment. The article authored by STEVENS P and published in the Kidney International in 2024 ranked first (8) with a total of 3, 525 citations and the highest annual citation rate. The second most cited study was published by NDUMELE C in Circulation in 2023 (9), accumulating 1, 238 citations. The third-ranked publication was the study by NDUMELE C in Circulation in 2023 (2), which received 816 citations.

**Table 4:** The top 10 high-cited papers in cardiovascular-kidney-metabolic syndrome related research from 2023 to 2025.

Rank	Paper	DOI	Total citations	TC per year	Normalized TC
1	STEVENS P, 2024, KIDNEY INT	10.1016/j.kint.2023.10.018	3, 525.00	1, 175.00	106.31
2	NDUMELE C, 2023, CIRCULATION	10.1161/CIR.0000000000001184	1, 238.00	309.50	3.74
3	NDUMELE C, 2023, CIRCULATION-a	10.1161/CIR.0000000000001186	816.00	204.00	2.46
4	MARTIN S, 2025, CIRCULATION	10.1161/CIR.0000000000001303	757.00	378.50	143.66
5	KHAN S, 2024, CIRCULATION	10.1161/CIRCULATIONAHA.123.067626	720.00	240.00	21.71
6	KHAN S, 2023, CIRCULATION	10.1161/CIR.0000000000001191	491.00	122.75	1.48
7	AGGARWAL R, 2024, JAMA	10.1001/jama.2024.6892	303.00	101.00	9.14
8	NEELAND I, 2024, NAT REV DISEASE PRIM	10.1038/s41572-024-00563-5	289.00	96.33	8.72
9	JOYNT M K, 2024, CIRCULATION	10.1161/CIR.0000000000001256	269.00	89.67	8.11
10	RYAN D, 2024, NAT MED	10.1038/s41591-024-02996-7	229.00	76.33	6.91

### 3.6 Keyword co-occurrence, clusters, and burst

**Figure 8:**
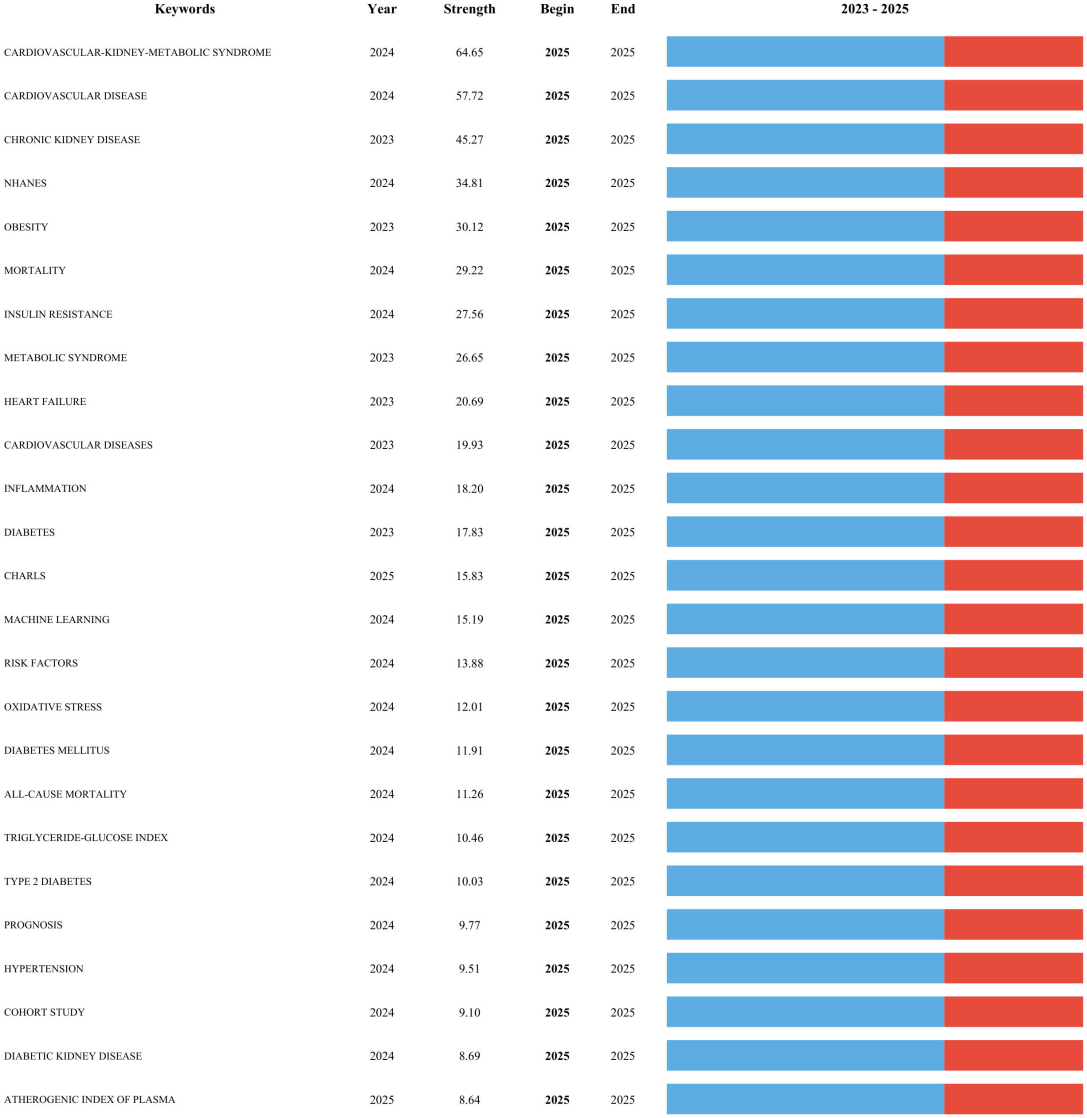
Top 10 keywords with the strongest citation bursts in the studies of CKM.

In **Figure 8**, the blue line represents the timeline, while the red segments indicate burst detections, reflecting the beginning, ending year, and duration of each keyword burst. Burst analysis was performed to identify emerging hotspots and evolving research trends in the field of CKM. Early burst keywords were primarily related to the newly introduced CKM framework, including cardiovascular-kidney-metabolic syndrome, CKM, and chronic kidney disease, reflecting the initial focus on defining the concept and its clinical components. Subsequently, keywords such as obesity, heart failure, mortality, integrated care, and cardiovascular disease emerged, indicating an expansion of research toward disease burden and clinical management. More recent burst keywords, including implementation, prevention, cardiovascular health, and GLP-1 receptor agonist, remained active through 2025, suggesting that these topics represent the latest research frontiers in the CKM field.

**Figure 9:**
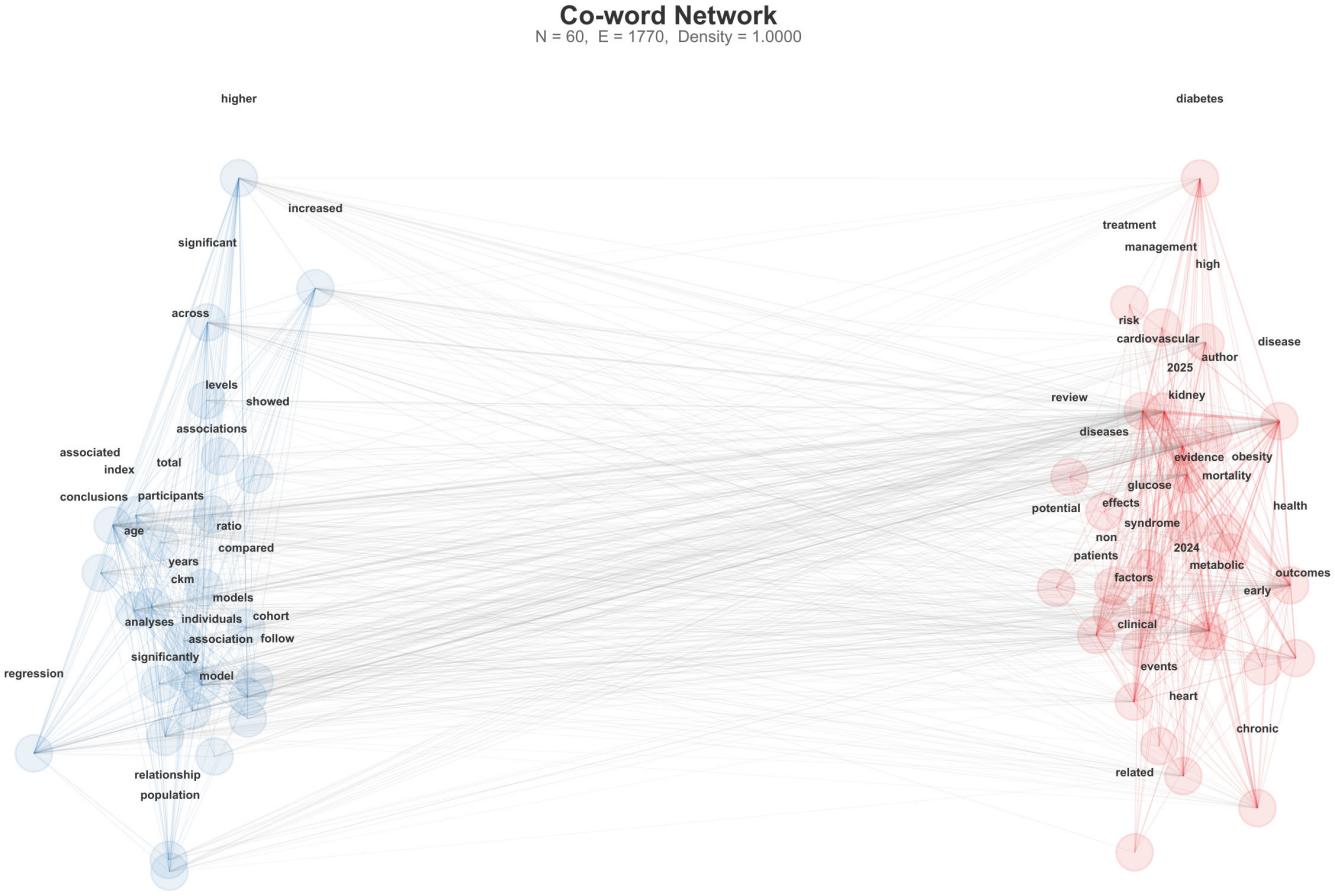
Analysis of keywords.

As shown in **Figure 9**, the keyword co-occurrence network was organized into several closely connected thematic clusters. One major cluster primarily focused on cardiovascular disease, heart failure, mortality, and outcomes, reflecting research related to disease prognosis and adverse clinical events. Another cluster was mainly associated with obesity, diabetes, glucose, kidney disease, and metabolic syndrome, highlighting the important role of metabolic dysfunction and renal impairment in CKM progression. Additional high-frequency terms, including “risk, “ “association, “ “management, “ “treatment, “ and “cohort, “ indicate that current studies increasingly emphasize risk stratification, preventive strategies, and clinical management of CKM. Overall, the co-occurrence network demonstrates that CKM research has evolved toward multidisciplinary and integrated approaches combining cardiovascular, renal, and metabolic perspectives.

### 3.7 References with the strongest citation bursts

**Figure 10:**
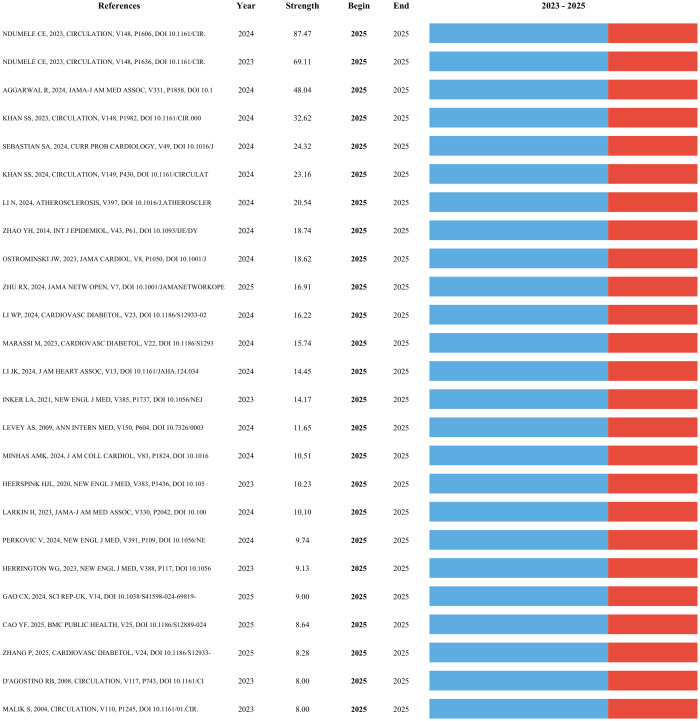
Top 10 most cited references with strong citation burstiness (MD = 2). The red bars mean some references are cited frequently; the blue bars represent references cited infrequently.

Citation burst analysis was performed using CiteSpace to identify references that attracted rapidly increasing scholarly attention during specific periods. As shown in **Figure 10**, the top 25 references with the strongest citation bursts were identified, and the red segments indicate the periods during which these publications experienced notable increases in citation frequency, reflecting their academic influence and emerging importance within the CKM research field.

Among the identified references, the guideline article published by Ndumele CE in Circulation in 2023 demonstrated the strongest citation burst (9), with a burst strength of 87.47 extending from 2024 to 2025. Another influential publication by the same author in Circulation in 2023 ranked second (2), with a burst intensity of 69.11 during the same period. In addition, the study by Aggarwal R published in JAMA in 2024 showed a strong citation burst of 48.04 (10). Several other highly cited studies, including those authored by Khan SS, Sebastian SA, and Ostrominski JW, also exhibited prominent burst signals. These influential publications mainly focused on the definition, epidemiology, risk stratification, and integrated management of CKM syndrome, highlighting the rapidly growing interest in multidisciplinary approaches linking cardiovascular, renal, and metabolic disorders. Furthermore, highly cited studies published in leading journals such as New England Journal of Medicine, JAMA Cardiology, and Cardiovascular Diabetology indicate increasing attention to clinical outcomes, preventive strategies, and evidence-based interventions in CKM research.

## 4 Discussion

This bibliometric analysis offers a comprehensive overview of the rapidly evolving research landscape surrounding CKM since its formal introduction in 2023. Although the concept is relatively recent, CKM research has experienced remarkable growth within just three years, reflecting strong scientific and clinical interest in this novel framework. Unlike traditional disease-centered models that address cardiovascular disease, chronic kidney disease, and metabolic disorders separately, the CKM paradigm emphasizes shared biological mechanisms and interconnected risk pathways across multiple organ systems. The rapid increase in publications observed in this study suggests that the CKM framework has gained wide acceptance as a promising approach to understanding and addressing the growing burden of chronic non-communicable diseases.

Several factors may explain the rapid growth of CKM-related research in a short span of time. To begin with, CKM was introduced as a unifying framework to address the increasing convergence of cardiovascular disease, chronic kidney disease, obesity, and diabetes—conditions that frequently coexist in aging populations around the world. By reclassifying these historically separated disorders into a single disease continuum, the CKM model has drawn substantial attention from the fields of cardiology, nephrology, endocrinology, and public health (11). Second, the rising burden of multimorbidity has exposed the limitations of organ-centered approaches and intensified the need for more comprehensive risk assessment and integrated management strategies (12). Finally, the accumulation of evidence highlighting the strong interrelationships among metabolic dysfunction, cardiovascular outcomes, renal impairment, and mortality has further reinforced the value of this framework, contributing to its rapid adoption across cardiology, nephrology, endocrinology, and public health research (13).

A notable finding of this study is the dominant contribution of China and the United States to the development of CKM research. The United States has played a central role in shaping the conceptual framework and establishing international collaborative networks, largely driven by major academic societies, large cohort studies, and clinical guideline initiatives. Meanwhile, China has emerged as the leading contributor in terms of publication volume, highlighting its growing influence in cardiovascular, metabolic, and aging-related research. This trend likely reflects substantial national investments in chronic disease prevention, large-scale population cohorts, electronic health databases, and translational medicine platforms. The increasing involvement of Chinese researchers is particularly significant given the country’s rapidly aging population and the escalating burden of obesity, diabetes, chronic kidney disease, and cardiovascular disease (14). Furthermore, the strong collaborative relationships observed between Chinese and Western institutions may facilitate the generation of globally applicable evidence and promote harmonization of CKM-related research methodologies (15). The observed deviation from Lotka’s law, particularly the higher-than-expected proportion of single-publication authors, likely reflects the emerging nature of CKM research. Since the CKM framework was formally introduced only in 2023, many investigators have recently entered the field, whereas only a limited number of research groups have developed sustained publication activity. As the discipline matures, a more stable core group of highly productive authors may gradually emerge.

The prominence of keywords such as mortality, heart failure, obesity, diabetes, and chronic kidney disease reflects the core clinical priorities of the CKM framework. These conditions represent interconnected components of the CKM spectrum and are major contributors to global morbidity and mortality. Their frequent occurrence suggests that current research has shifted from studying isolated cardiovascular, renal, or metabolic disorders toward integrated risk assessment and multidisciplinary management. This trend is consistent with the CKM framework, which emphasizes early prevention, comprehensive risk stratification, and coordinated care across cardiology, nephrology, endocrinology, and primary care.

Keyword and citation analyses indicate that CKM research has moved beyond a focus on simple disease coexistence toward a more integrated understanding of systemic pathophysiology. High-frequency keywords related to obesity, diabetes, kidney disease, heart failure, and mortality suggest that researchers increasingly view CKM as a multidirectional network of biological dysfunction rather than a collection of isolated diseases. Recent evidence further suggests that vascular aging represents a fundamental biological process linking the cardiovascular, kidney, and metabolic components of CKM syndrome (16). Beyond traditional risk factors, arterial stiffness and accelerated vascular aging may contribute to endothelial dysfunction, chronic inflammation, and progressive multiorgan impairment, reinforcing the concept of CKM as a systemic rather than organ-specific disorder. These findings further support the multidirectional pathophysiological interactions highlighted by the current keyword evolution analysis. Emerging concepts such as inflammation, insulin resistance, adipose tissue dysfunction, endothelial injury, oxidative stress, and neurohormonal activation may represent common mechanistic pathways linking cardiovascular, renal, and metabolic abnormalities (17). More recently, the appearance of heart failure and outcome-related keywords indicates a growing focus on prognostic assessment and clinical translation (18). This evolution reflects a broader shift in medicine from disease treatment toward risk prediction, prevention, and long-term health optimization.

From both clinical and public health perspectives, the increasing emphasis on mortality and adverse clinical outcomes is particularly important. Emerging evidence also indicates that CKM syndrome is highly heterogeneous. Data-driven cluster analyses have identified distinct CKM phenotypes with different cardiometabolic profiles and prognostic trajectories, suggesting that individualized risk stratification may improve clinical decision-making (19). In parallel, modifiable health behaviors captured by the American Heart Association Life’s Essential 8 metrics have been associated with improved prognosis among patients with advanced CKM syndrome, emphasizing the importance of lifestyle optimization (20). Furthermore, recent longitudinal evidence has highlighted the potential value of dynamic biomarkers such as remnant cholesterol in monitoring CKM progression, suggesting that temporal changes rather than single baseline measurements may better reflect disease evolution (21). Accumulating evidence suggests that progression through CKM stages is associated with substantially increased risks of all-cause mortality, cardiovascular mortality, hospitalization, disability, and healthcare utilization (22). Importantly, these risks often emerge years before the onset of overt cardiovascular events. As a result, CKM may provide a valuable framework for identifying high-risk individuals at earlier stages of disease development. Early recognition of metabolic dysfunction, obesity, impaired kidney function, and subclinical cardiovascular abnormalities could allow timely implementation of preventive strategies before irreversible organ damage occurs (23). Such an approach aligns closely with the principles of precision prevention and life-course disease management.

The emergence of expanded concepts such as cardiovascular-kidney-liver-metabolic syndrome further illustrates the ongoing evolution of this research field. Growing evidence indicates that metabolic dysfunction-associated steatotic liver disease is closely linked to cardiovascular disease, chronic kidney disease, insulin resistance, and systemic inflammation (24). The inclusion of hepatic involvement highlights the possibility that future research may increasingly focus on multi-organ interactions rather than traditional organ-specific disease classifications (25). This shift may fundamentally reshape how chronic diseases are conceptualized, diagnosed, and managed in both clinical practice and population health settings.

From a health-economic perspective, the implications of CKM are substantial. The coexistence of cardiovascular, renal, and metabolic disorders is associated with high rates of hospitalization, medication use, disability, and premature mortality, generating considerable healthcare expenditures worldwide (26). As populations continue to age and multimorbidity becomes increasingly common, the economic burden attributable to CKM is expected to rise substantially (27). Early identification and intervention may therefore yield significant long-term cost savings by preventing disease progression and reducing downstream complications. Future economic evaluations should investigate the cost-effectiveness of integrated CKM screening programs, multidisciplinary management models, and novel pharmacological interventions that simultaneously target multiple disease pathways. The clinical applicability of the CKM framework has also expanded rapidly in recent years. Population-based studies have demonstrated an increased prevalence of CKM syndrome during the COVID-19 pandemic, highlighting the vulnerability of individuals with multiple cardiometabolic conditions to large-scale public health crises (28). Meanwhile, intervention studies suggest that short-term comprehensive management strategies integrating lifestyle modification and metabolic optimization can significantly improve CKM status and several of its individual components in patients with type 2 diabetes (29). Together, these findings indicate that CKM is evolving from a conceptual framework toward a clinically actionable model for integrated prevention and management.

Beyond the traditional focus on cardiovascular, renal, and metabolic outcomes, our bibliometric findings also suggest that CKM research is gradually expanding toward a more comprehensive, patient-centered framework. Although mental health-related terms have not yet emerged as dominant keywords, recent studies have increasingly explored the associations between CKM and depression, anxiety, social connectedness, cognitive function, and other neuropsychological outcomes (30). This shift reflects growing recognition that psychological and behavioral factors may influence the entire CKM continuum through multiple pathways, including chronic inflammation, neuroendocrine dysregulation, lifestyle behaviors, medication adherence, and healthcare utilization (31, 32). Conversely, the substantial symptom burden and long-term treatment demands associated with CKM may further increase the risk of adverse mental health outcomes, creating a bidirectional relationship that may accelerate disease progression (33). These emerging findings indicate that future CKM research is likely to move beyond organ-specific risk assessment toward integrated models incorporating mental health, behavioral medicine, and social determinants of health. Such a multidisciplinary approach may facilitate earlier identification of high-risk individuals, improve patient-centered management, and ultimately enhance long-term cardiovascular, renal, and metabolic outcomes.

Several policy implications arise from these findings. First, CKM screening should be incorporated into routine primary care and public health programs, particularly among individuals with obesity, diabetes, hypertension, or chronic kidney disease. Second, risk assessment tools capable of simultaneously evaluating cardiovascular, renal, and metabolic health need further development and validation. Third, healthcare systems may benefit from multidisciplinary care models that promote collaboration among cardiologists, nephrologists, endocrinologists, hepatologists, nutrition specialists, and primary care physicians. Finally, public health strategies should prioritize modifiable risk factors including unhealthy diet, physical inactivity, obesity, smoking, and poor metabolic control, which represent common upstream drivers of CKM progression.

Future research is likely to focus increasingly on precision medicine, digital health, and translational implementation. Artificial intelligence, machine learning algorithms, wearable devices, and electronic health record integration may facilitate earlier detection of high-risk individuals and improve dynamic risk prediction (34). Multi-omics technologies may further clarify the biological mechanisms linking cardiovascular, renal, metabolic, and hepatic dysfunction (35). In addition, large longitudinal cohorts and real-world databases will be essential for evaluating long-term outcomes, disease trajectories, and intervention effectiveness across diverse populations (36). As the CKM framework continues to mature, future studies should move beyond descriptive epidemiology toward mechanistic investigations, implementation science, and intervention-based research aimed at improving patient outcomes. Recent comprehensive reviews have further emphasized that future CKM research should integrate epidemiology, mechanistic investigations, precision medicine, digital health technologies, and multidisciplinary management strategies (37). Our bibliometric findings strongly support this trend, as implementation science, prevention, artificial intelligence, and integrated care emerged among the most active research frontiers.

Several limitations should be acknowledged. As with all bibliometric studies, the findings are influenced by database selection and coverage, indexing practices, and citation behaviors, potentially underrepresenting recently published studies because of citation lag and excluding non-English literature. Furthermore, the relatively short time since the introduction of CKM may limit the stability of citation-based indicators. In addition, because CKM was only formally introduced in 2023, publication-based growth indicators may be sensitive to the small number of studies available during the initial stage of field development. Therefore, growth rates should be interpreted cautiously. Citation-based metrics may also be affected by self-citation and cannot directly evaluate the methodological quality or clinical significance of individual studies. Moreover, the results of network visualization and clustering may vary depending on software algorithms and parameter settings, although standardized analytical procedures were applied in this study. Nonetheless, this study provides a timely overview of the intellectual structure, collaborative landscape, emerging hotspots, and future directions of CKM research. The findings demonstrate that CKM has rapidly evolved into a highly active and multidisciplinary field with important implications for clinical practice, public health policy, healthcare economics, and future chronic disease prevention. strategies.

## 5 Conclusion

This bibliometric analysis reveals that CKM research has grown quickly since its debut in 2023, driven largely by China and the United States. Global publication output is rising, and international collaboration is strong. The field is now moving toward integrated studies on cardiometabolic-renal interactions and patient outcomes. However, CKM research is still at an early stage. Future work should strengthen interdisciplinary partnerships and boost clinical translation to better support risk stratification and prevention.

## Data Availability

The original contributions presented in the study are included in the article/supplementary material. Further inquiries can be directed to the corresponding author.
